# Short chain fatty acids produced by colonizing intestinal commensal bacterial interaction with expressed breast milk are anti-inflammatory in human immature enterocytes

**DOI:** 10.1371/journal.pone.0229283

**Published:** 2020-02-21

**Authors:** Nan Zheng, Yanan Gao, Weishu Zhu, Di Meng, W. Allan Walker

**Affiliations:** 1 State Key Laboratory of Animal Nutrition, Institute of Animal Sciences, Chinese Academy of Agricultural Sciences, Beijing, China; 2 Mucosal Immunology and Biology Research Center, Massachusetts General Hospital for Children, Harvard Medical School, Boston, Massachusetts, United States of America; Southern Illinois University School of Medicine, UNITED STATES

## Abstract

Necrotizing enterocolitis (NEC) is a devastating intestinal emergency that affects ten percent of very low birth weight premature babies and costs society in both expense and heartache. It is probably caused by an inappropriate interaction of colonizing bacteria with an immature intestine. A possible preventative measure is to feed prematures their mother’s expressed breast milk in conjunction with a probiotic. This synbiotic prevention reduces the severity and incidence of this condition. This study was designed to determine the mechanism of the synbiotic effect in human and mouse fetal intestine. Breast milk interacting with a NEC preventative probiotic such as *Bifidobacterium infantis* can produce increased levels of short chain fatty acids (acetate, propionate and butyrate) (SCFAs). SCFAs are known to be anti-inflammatory in mature enterocytes and immunocytes. Very little is known about their role in immature intestine. When exposed to a human fetal cell line, fetal intestinal organoids and fetal mouse intestine, these SCFAs were anti-inflammatory. Their mechanism of anti-inflammation differed from those reported for mature cells by involving the G-protein coupled receptor (GPR 109A) and inhibiting histone deacetylase 4 and 5. These bacterial metabolites may help explain the synbiotic anti-inflammatory effect of breast milk and probiotics given to premature infants at risk for NEC.

## Introduction

Necrotizing enterocolitis (NEC) is an inflammatory necrosis of the distal small intestine and colon that commonly affects very premature infants (10% incidence) less than 1500grams in birthweight [1]. We have reported that its pathogenesis is in part due to an inappropriate reaction of colonizing bacteria with an immature intestine [2]. For example, colonizing bacteria (both pathogens and commensals) can create an excessive inflammatory response in the immature intestine rather than developing immune homeostasis [3]. This excessive inflammatory response may be due to an immature innate reaction to the colonizing bacteria [2,4] because the immature intestine expresses excessive toll-like receptor-4 (TLR4) on its surface and increased Nuclear Factor κB (NFκB) and Interleukin-8 (IL-8) and has less expression of inflammatory regulators single Ig IL-1-related receptor (SIGRR), IL-1receptor-associated kinase M (IRAK-M), tumor necrosis factor, alpha-induced protein3 or A-20, etc. [2,5].

Although the pathogenesis of NEC is incompletely understood, clinical studies have suggested that feeding prematures their mother’s expressed breast milk or giving them probiotics may prevent or lessen the severity of the disease [6,7]. A recent study suggests that prevention is most effective when breast milk and probiotics are given together (synbiotic effect) [8].

Short chain fatty acids (SCFAs) are produced by intestinal commensal bacteria interacting with a diet rich in complex carbohydrates which cannot be metabolized by enzymes in the small intestine [9]. In the immature human intestine, SCFAs are produced when expressed breastmilk fed to prematures interacts with colonizing bacteria [10,11] for example, Bifidobacteria infantis can produce increased levels of SCFAs (acetate, propionate and butyrate) [12]. A number of studies in mature intestine have shown that SCFAs have anti-inflammatory effects under conditions of intestinal inflammation such as dextran sulfate sodium (DSS) colitis and are in short supply in patients with inflammatory bowel disease and rheumatoid arthritis [13–15]. Histone post-translational modifications are fundamental regulators of gene expression and are tightly controlled by histone deacetylases (HDACs). It is reported that SCFAs modulate immune and inflammatory responses via activation of their receptors free fatty acid receptors type 2 and3 (FFAR2 and FFAR3) and G protein-coupled receptor 109A (GPR109A) to inhibit HDACs [16–18]. In this study, we determined if SCFAs (acetate, propionate and butyrate) were anti-inflammatory in a fetal cell line, in fetal organoids and in fetal mouse intestine after an inflammatory stimulus with IL-1β. We also have begun to determine whether SCFAs receptors and HDACs are involved in the anti-inflammatory enterocyte cellular mechanism for this response in immature enterocytes compared to that reported for mature enterocytes [17,19].

Accordingly, this study was designed to determine how the synbiotic effect works by determining the interaction of bacterial metabolites SCFAs on immature intestinal inflammation.

## Results

### SCFAs inhibit IL-1β-induction of IL-8 secretion in immature enterocytes (H4 cells)

**Fig 1:**
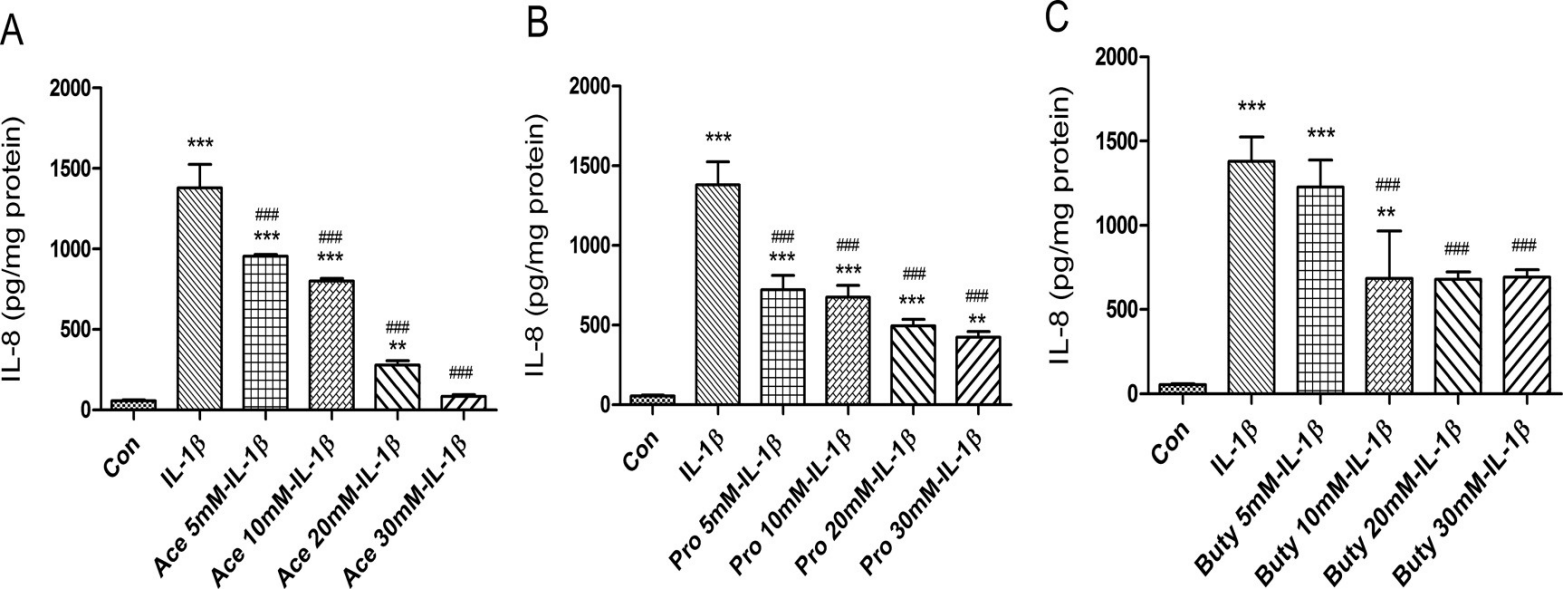
The effects of different doses of short-chain fatty acids (SCFAs) on prevention of IL-1β-induced IL-8 induction in H4 cells. H4 cells were pretreated with or without SCFAs, **(A)** Acetate (5−30 mM), **(B)** Propionate (5−30 mM), and **(C)** Butyrate (5−30 mM) for 30 min before exposure to IL-1β (1ng/ml) for 24 h. The secretion of IL-8 into the supernatants was determined by enzyme-linked immune sorbent assay (Elisa). The results are represented as the mean ± SEM of three independent experiments. One -way ANOVA and Tukey post hoc tests were used for statistic, n = 3. Differences compared to the control group were considered significant at **p<0.01, *** p<0.001; differences compared to the IL-1β group were considered significant at ### p<0.001. Acetate, Ace; propionate, Pro; butyrate, Buty.

To investigate if SCFAs have an anti-inflammatory effect on immature intestinal epithelial cells, H4 cells were pretreated with different doses of SCFAs before an IL-1β stimulation. A range of doses of acetate **(Fig 1A**) and propionate **(Fig 1B**) from 5 mM to 30 mM significantly inhibited IL-1β-induced IL-8 secretion in a dose dependent manner. However, 5mM of butyrate had no inhibitory effect but the other doses (10mM, 20mM and 30mM) had similar inhibitory effects, suggesting that no dose dependent response was observed with butyrate **(Fig 1C**).

### SCFAs have an anti -inflammatory effect on fetal intestinal organoids

**Fig 2:**
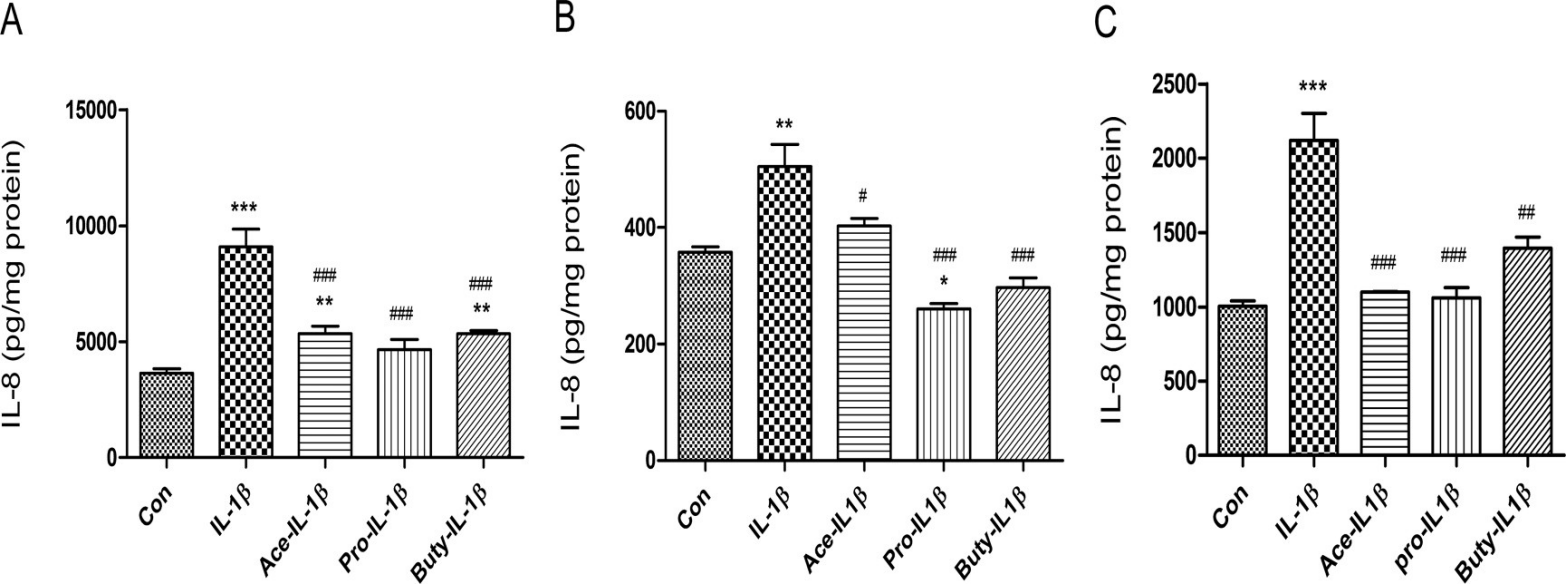
SCFA inhibited IL-1β induced IL-8 secretion in H4 organoids and other fetal human intestinal organoids. H4 organoids **(A),** and fetal human small intestinal organoids from gestational age 15-week **(B)** and 22-week **(C)** cultured as a monolayer in trans-well plates were pretreated with 20 mM of acetate, propionate or butyrate for 30 min before IL-1β stimulation (1ng/ml for 24h). The secretion of IL-8 into the supernatants was measured by Elisa. Data are represented as the mean ± SEM of three independent experiments. One -way ANOVA and Tukey post hoc tests were used for statistic, n = 3. Differences compared to the control group were considered significant at*p<0.05, **p<0.01, *** p<0.001; differences compared to the IL-1β group were considered significant at # p<0.05, ### p<0.001.

H4 cells, a 2-dimensional non-polarized immature intestinal monolayer, can only be treated from the surface side. However, an inflammatory infection of immature enterocytes may occur from both the luminal or basolateral side. Based on ours and other’s observations that the 3D intestinal organoid can be cultured in transwells to form 3D monolayers of functional polarized epithelial cells [20], a monolayer derived from H4 organoids was used to investigate if SCFA can protect the inflammatory cytokines from all sides. Monolayers of H4-organoids were pretreated with SCFAs (20 mM, 30 min) from the apical side and then treated with IL-1β (1ng/ml, 24h) on the basolateral side (**Fig 2A**). We found that IL-1β significantly induced IL8 induction (***p<0.001), and SCFAs (acetate, propionate and butyrate) significantly inhibited IL-1β-induced-IL-8 secretion (vis IL-1β ###p<0.001 in three SCFAs), the same results noted in the H4 cell line.

When additional human fetal organoids [age 15 weeks (**Fig 2B**) and 22 weeks (**Fig 2C**)] were exposed to IL-1β, the levels IL8 were increased significantly (***p<0.001). However, if we pretreated them with individual SCFAs at levels shown to be anti-inflammatory in H4 cells and organoids, a significant anti-inflammatory effect was noted after IL-1β stimulation confirming the SCFAs generalized anti-inflammatory effect in immature human intestine.

### SCFAs have an anti-inflammatory effect in fetal mouse intestinal organ cultures

**Fig 3:**
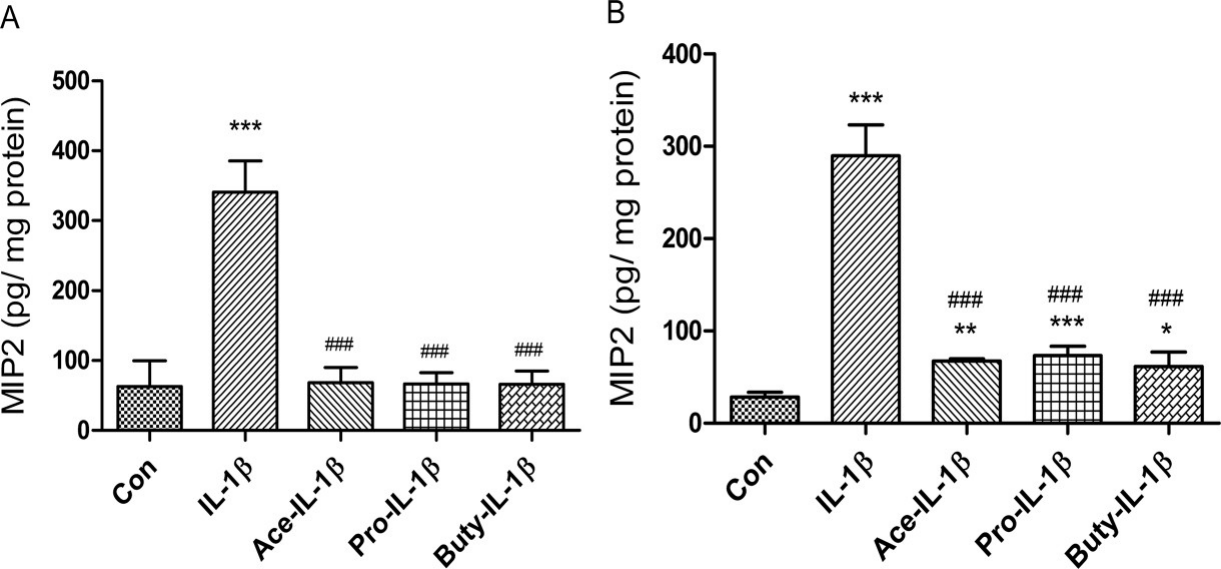
The anti-inflammatory effects of SCFAs on immature enterocytes in ex vivo mouse intestine. Embryonic 18.5day (E 18.5) C57BL6 fetal mouse small (**A**) and large (**B**) intestinal organ culture were pretreated with SCFAs (20 mM for 30 min) before IL-1β stimulation (1ng/ml for 24h). The secretion of IL-8 into the supernatants was assayed by Elisa. Data are represented as the mean ± SEM of two independent experiments. N = 6 for each experiment. One -way ANOVA and Tukey post hoc tests were used for statistic. Differences compared to the control group were considered significant at **p<0.01, *** p<0.001; differences compared to the IL-1β group were considered significant at ### p<0.001.

To investigate if SCFAs have an anti-inflammatory effect on fetal mouse intestinal epithelium, C57BL6 fetal mouse (E 18.5 d) small intestine (**Fig 3A**) and colon (**Fig 3B**) organ cultures [21,22] were subjected to 20 mM of SCFAs (acetate, propionate and butyrate) before an IL-1β stimulation. The results showed that all three SCFAs significantly reduced IL-1β-induced macrophage inflammatory protein2 (MIP2), a murine homologue of IL8, secretion in fetal mouse small intestine and colon ex-vivo organ cultures confirming the anti-inflammatory effect of SCFAs on immature mouse intestine.

### SCFAs inhibit IL-1β- induction of HDAC3 and HDAC5 genes in immature but not mature enterocytes

**Fig 4:**
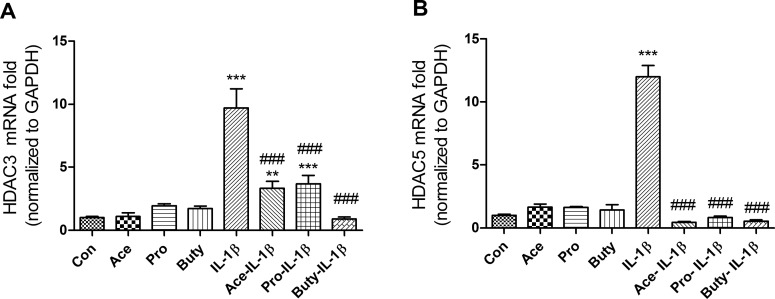
SCFAs inhibit IL-1β induced histone deacetylases HDAC3 and HDAC5 mRNA expression in immature enterocytes. H4 cells **(A-B)** were pretreated with 20 mM of SCFAs for 30 min before IL-1β stimulation (1ng/ml for 4h). HDAC3 **(A)** and HDAC5 **(B)** mRNA expression were determined by real time RT-PCR. Data are represented as the mean ± SEM of three independent experiments. One -way ANOVA and Tukey post hoc tests were used for statistic, n = 3. Differences comparison to the control group were considered significant at **p<0.01, *** p<0.001; differences comparison to the IL-1β group were considered significant at ### p<0.001.

**Fig 5:**
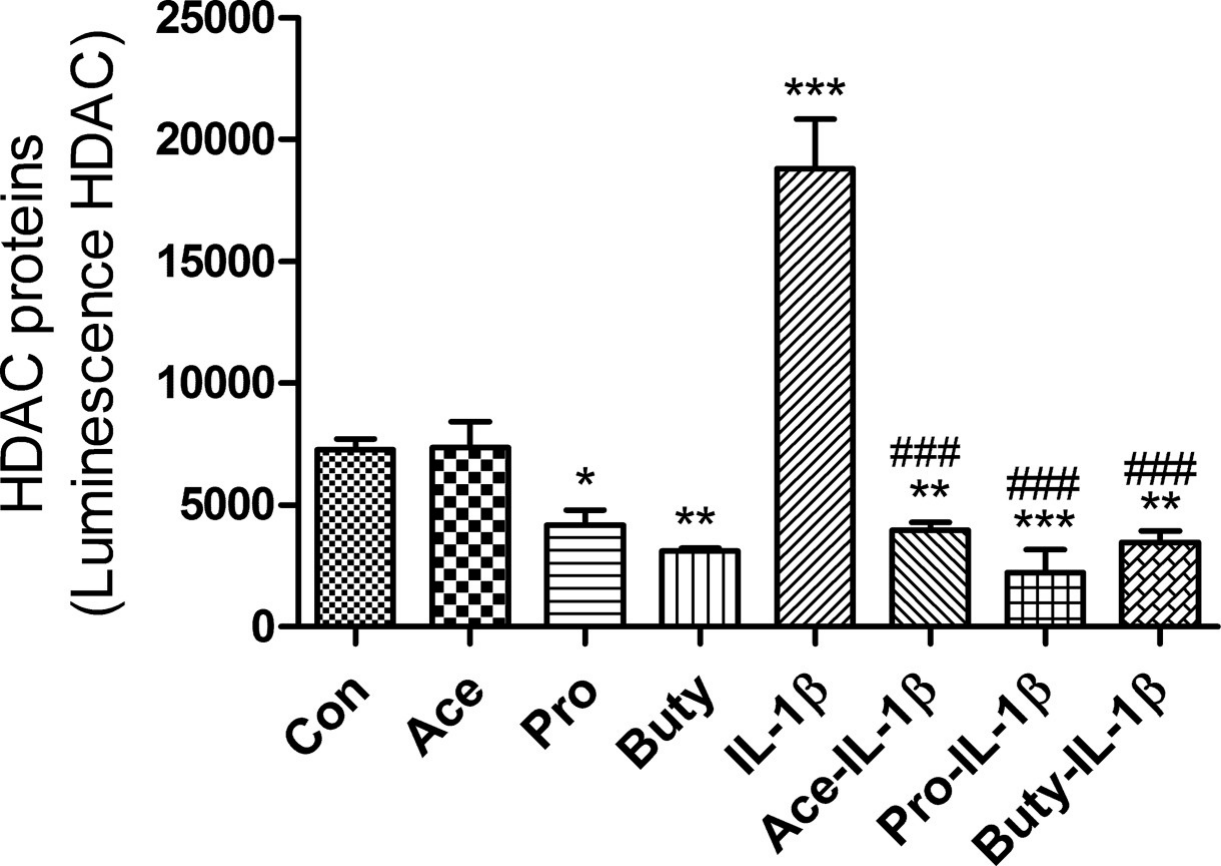
SCFAs inhibit IL-1β induced HDAC protein expression in immature enterocytes. H4 cells were pretreated with 20 mM of SCFAs for 30 min before IL-1β stimulation (1ng/ml) for 24h. Total HDACs protein (including HDAC3 and HDAC5) level was determined by Luminescent assay (using HDAC-Glo^™^ I/II Assay kit). Data are represented as the mean ± SEM of two independent experiments. One -way ANOVA and Tukey post hoc tests were used for statistic, n = 3. Differences comparison to the control group were considered significant at **p<0.01, *** p<0.001; differences comparison to the IL-1β group were considered significant at ### p<0.001.

Many signaling pathways cause a cellular response that involves a change in gene expression. HDACs are a class of enzymes that remove acetyl groups from an acetyl -lysine amino acid on a histone allowing the histones to wrap the DNA more tightly and activate a series of gene expressions. To investigate if SCFAs anti IL-1β-induced inflammation was involved in regulation of HDACs signaling pathways in epithelial cells, immature enterocytes (H4 cells) and mature enterocytes (Caco2 cells) were pretreated with SCFAs before IL-1β stimulation followed by a determination of HDAC gene expression. We found that three SCFAs alone had no effect on the mRNA expression of HDAC3 or HDAC5, but all of them can inhibit IL-1β-induced HDAC3 and HDAC5 mRNA in H4 cells (**Fig 4A and 4B**) but not in Caco2 cells (**S1B and S1C Fig**), although these three SCFAs can inhibit IL-1β-induced IL-8 secretion in Caco2 cells (**S1A Fig**), suggesting that a different anti-inflammatory mechanism exists between immature and mature intestinal epithelial cells. These results indicate that SCFAs anti IL-1β-induced HDACs gene induction only occurred in immature enterocytes. To provide additional evidence that IL-1β induced HDACs induction can be regulated by SCFAs, HDACs protein was determined by a luciferase assay (**Fig 5)**. As expected, IL-1β-induced HDACs protein induction can also be inhibited by pretreatment with SCFAs. In addition, propionate and butyrate alone (not acetate alone) can inhibit the HDACs protein expression as well.

### HDACs mediate IL-1β-induced IL-8 secretion in immature enterocytes

**Fig 6:**
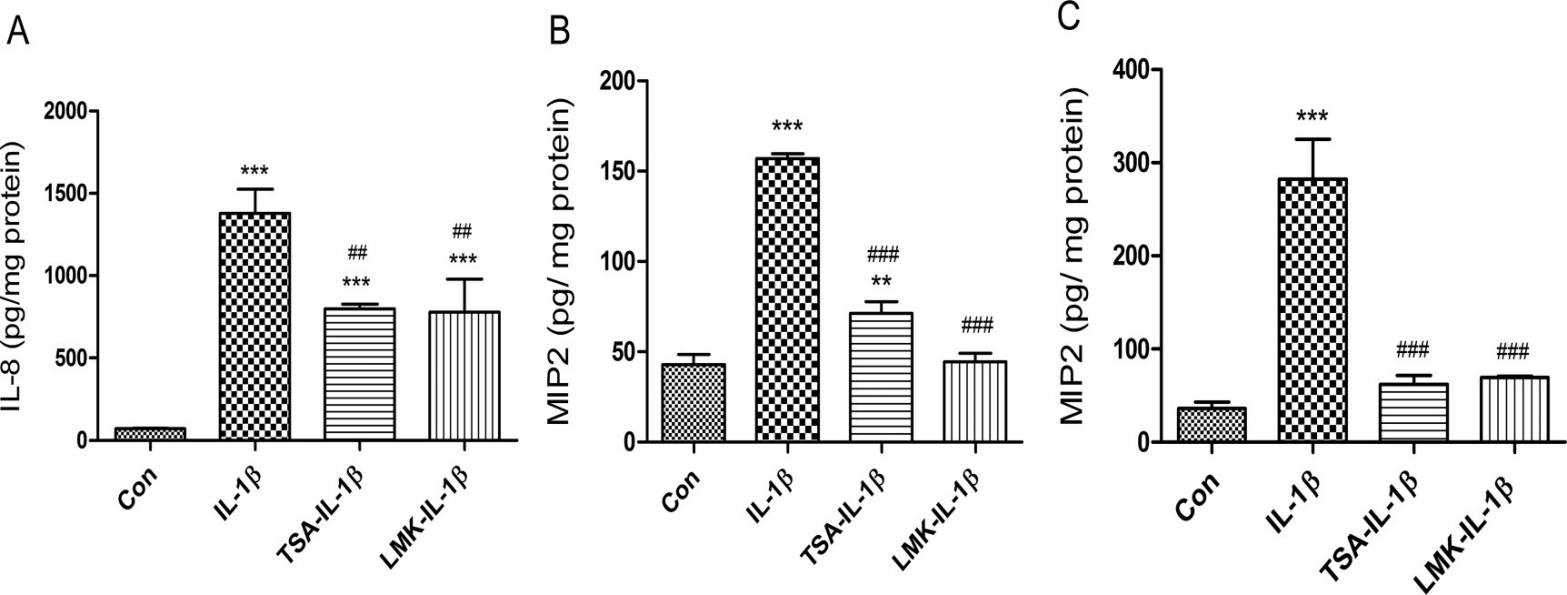
Both HDAC3 and HDAC5 are required for mediating IL-1β induced IL-8 secretion in immature enterocytes *in vitro* and *ex vivo*. H4 cells **(A)**, C57BL6 fetal mouse small **(B)** and large intestinal **(C)** organ cultures were pretreated with HDAC3 inhibitor Trichostatin A (TSA) 100nM or HDAC4-5 inhibitor LMK-235 (LMK) 100nM for 30 min before IL-1β stimulation (1 ng/mL for 24 h). The secretion of IL-8 into the supernatants was assayed by Elisa. Data are represented as the mean ± SEM of three independent experiments for H4 cells and two independent experiments for mouse. One -way ANOVA and Tukey post hoc tests were used for statistic. n = 3 (H4 cells), n = 6 (mouse). Differences compared to the control group were considered significant at **p<0.01, *** p<0.001; differences compared to the IL-1β group were considered significant at ## p<0.01, ### p<0.001.

To provide additional evidence that HDACs signaling pathways are involved in IL-1β-induced IL-8 secretion in immature enterocytes, H4 cells and fetal mouse small and colonic intestinal organ cultures were pretreated with HDACs inhibitors [trichostain A (TSA, inhibits HDAC 1,3,4,6 and 10) and LMK325 (inhibits HDAC 4 and 5)] before IL-1β stimulation. The results showed that IL-1β lost its effect on IL-8 secretion in both TSA and LMK pretreated H4 cells (**Fig 6A)**, fetal mouse small intestinal (**Fig 6B)** and colonic (**Fig 6C)** organ cultures indicating that these HDACs mediate IL-1β-induced IL-8 secretion in immature enterocytes in vitro and ex-vivo.

### SCFAs activate G- protein coupled receptor 109A (GPR109A) but not GPR43 in response to IL-1β stimulation in immature enterocytes

**Fig 7:**
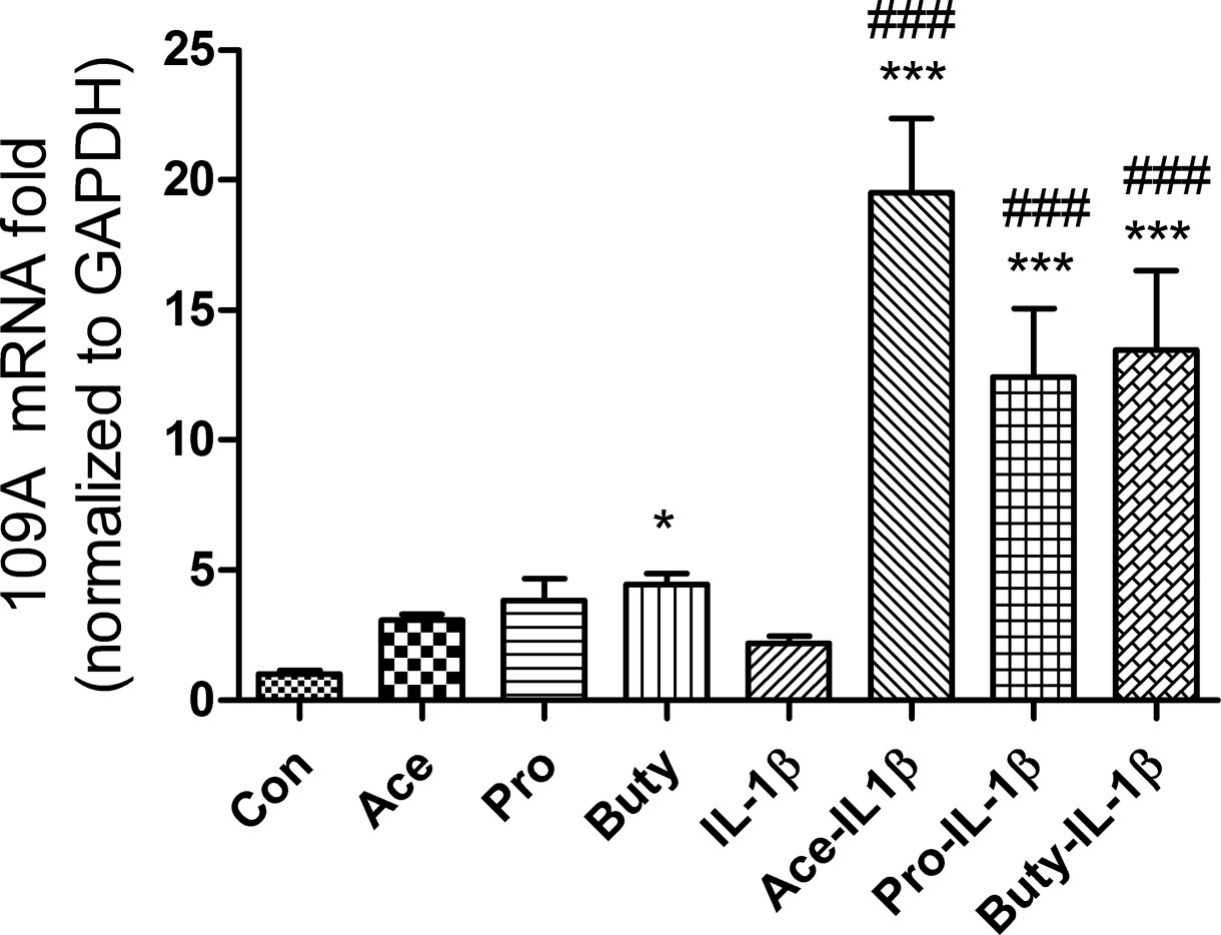
SCFAs increase G protein-coupled receptors 109A (GPR109A) mRNA expression in response to IL-1β stimulation in human immature enterocytes. H4 cells were pretreated with or without 20 mM of SCFAs for 30 min before IL-1β stimulation (1ng/ml for 4h); or treated with 20 mM of SCFAs alone. GPR109A mRNA levels were determined by real time RT-PCR. Data are represented as the mean ± SEM of three independent experiments, n = 3. One -way ANOVA and Tukey post hoc tests were used for statistic. Differences compared to the control group were considered significant at *** p<0.001; differences compared to the IL-1β group were considered significant at ### p<0.001.

Free Fatty Acid Receptor 2 (FFAR2) or GPR43 and, FFAR3 or GPR41 and GPR 109A are the three principal SCFAs receptors. To investigate if SCFAs activate these receptors in response to IL-1β stimulation in immature enterocytes, mRNA fold change of the genes coding these receptors were determined by real time PCR in H4 cells. We found that FFAR2, FFAR3 mRNA were present at very low levels and that no changes were observed after IL-1β stimulation in H4 cells. However, the GPR 109A mRNA was detectable and up-regulated to varying degrees was noted upon IL-1β stimulation with all three SCFAs pretreatment of H4 **(Fig 7)** cells. SCFAs alone affected GPR 109A mRNA levels in H4 cells (butyrate alone significantly increased 109A mRNA expression, acetate and propionate alone also increased 109A mRNA expression but not significantly). IL-1β alone did not induce GPR 109A mRNA expression in H4 cells.

### SCFAs lose their anti-inflammatory effect on IL-1β-induced IL8 secretion with a GPR 109A inhibitor in H4 cells

**Fig 8:**
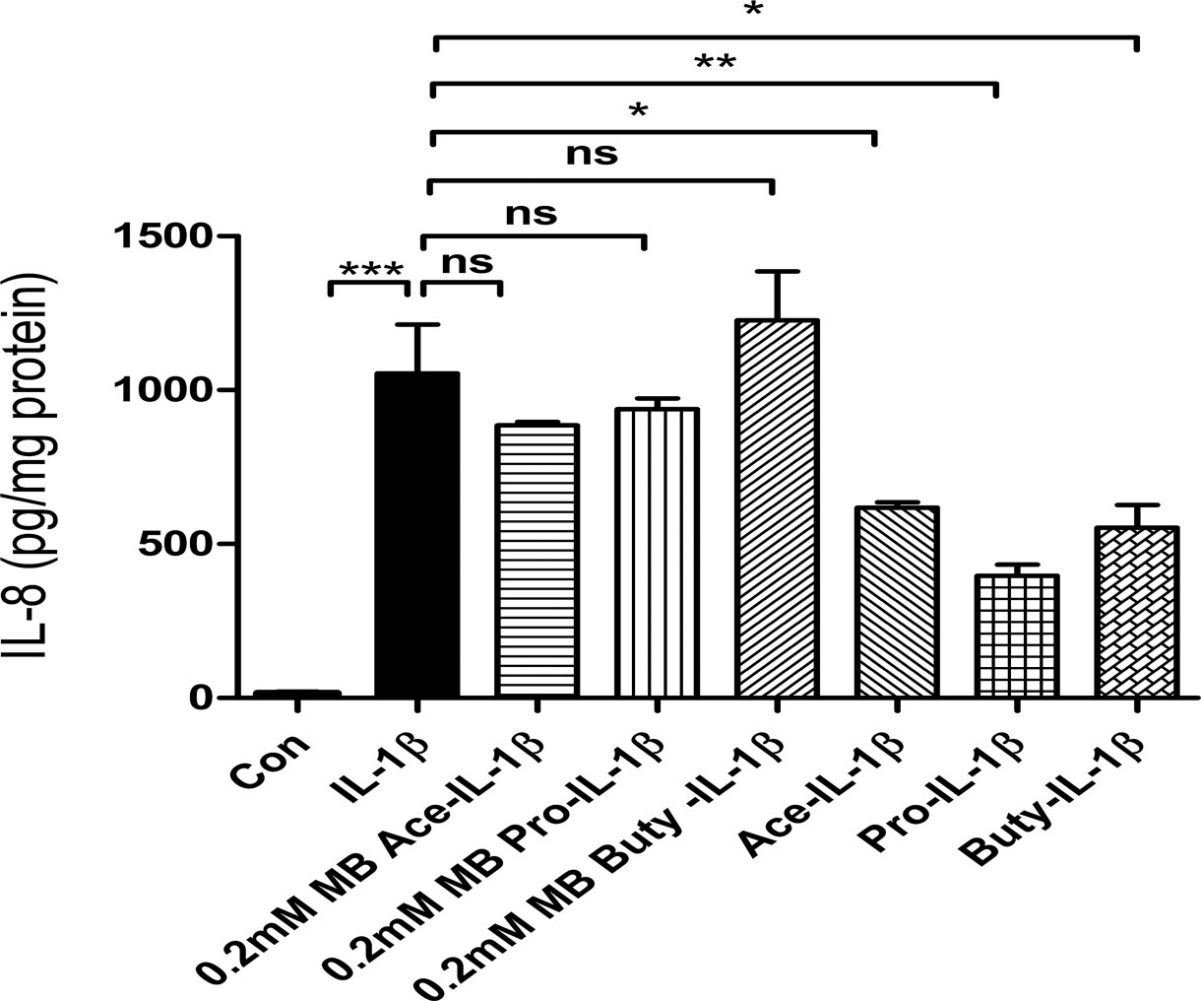
109A is required for SCFAs anti IL-1β induced IL8 induction in H4 cells. H4 cells were pretreated with 109A inhibitor mepenzolate bromide (MB) (0.2mM) for 30 minutes then treated with SCFAs before IL-1β stimulation. The secretion of IL-8 into the supernatants was assayed by Elisa. Data are represented as the mean ± SEM of three independent experiments. n = 3. One -way ANOVA and Tukey post hoc tests were used for statistic (*p<0.05, **p<0.01, *** p<0.001).

To further confirm that SCFAs regulates IL-1β-induced IL8 secretion via the GPR 109A receptor in H4 cells, an inhibitor of GPR 109A [0.2mM mepenzolate bromide (MB)] was used [23]. The results showed that SCFAs lost their anti-inflammatory effect on IL-1β-induced IL8 secretion in H4 cells **(Fig 8).**

## Discussion

Necrotizing enterocolitis (NEC) is the most serious intestinal emergency occurring in the premature [1]. Its pathogenesis is not completely understood but is related to colonizing bacteria interacting with an immature intestinal tract [2,24]. We have previously reported that the immature intestine favors excessive inflammation over immune homeostasis [3,4]. This occurs due to an innate immune inflammatory response which excessively reacts to both pathogens as well as commensal bacteria [3] because components of innate immunity (TLR4, NFκB and IL-8) are excessively expressed and factors that control the innate immune inflammation (SIGRR, IRAK-M, A20, etc.) are under expressed [2,5]. We believe that this immaturity contributes to the increased susceptibility of prematures to developing NEC.

Although to date no preventative measures have been established to routinely prevent the expression of NEC, certain approaches have reduced its incidence and severity. If a premature infant weighing less than 1500 grams is given its mother’s expressed breast milk, the premature is less likely to develop NEC and if it does the condition is less severe [11]. In addition, in small clinical trials certain probiotics have reduced the incidence and severity of NEC [7,8]. A recent study has shown that the use of expressed breast milk and a probiotic in combination (synbiotic effect) may be more effective than either treatment alone [8]. Accordingly, we have attempted to determine the mechanism by which the synbiotic approach is effective.

In other not yet published studies, we have shown that *Bifidobateria infantis* can metabolize tryptophan in breast milk to produce indole-lactic acid which is developmentally anti-inflammatory in a fetal human intestinal cell line, fetal organoids, fetal mouse intestine and fetal NEC enterocytes. The bacterial secreted metabolite interacts with the transcription factor aryl-hydrocarbon to prevent its transcription of IL-8 after an IL-1β inflammatory stimulus. This observation suggests a mechanism for the synbiotic anti-inflammatory effect of breastmilk and a probiotic in NEC [24,25].

In this study we examined another effect on inflammation of other bacterial metabolites produced after interaction with expressed breastmilk. It has been reported that intestinal colonizing bacteria can metabolize complex carbohydrates expressed in breastmilk but not digested by small intestinal enzymes to produce large quantities of SCFAs (acetate, propionate and butyrate) [11,26]. It has been shown that these SCFAs have immunologic activating effects on mature human enterocytes [9]. However, very little has been reported about their effect on immature human enterocytes.

In this study, IL-1β can stimulate the induction of IL8 in a human fetal enterocyte cell line (**Fig 1)**, organoid (from H4 Cells) (**Fig 2A**) and other human fetal enteroids (**Fig 2B and 2C**) and the induction of MIP2 (murine homologue of IL8) in fetal mouse intestine (**Fig 3**). Three short chain fatty acids have been shown to inhibit IL-1β induced IL8 or MIP2 induction significantly in all those models (**Figs 1–3**). IL-1β levels are known to be increased in the intestinal contents of patients with NEC [1,6]. This observation suggests that the synbiotic effect of feeding mothers expressed breast milk in combination with a probiotic such as *Bifidobacterium infantis* may represent a synbiotic mechanism for the protective approach in the premature infant.

It has previously been reported that SCFAs interact with the G protein coupled protein receptor (GPR) 43 in mature immune cells and enterocytes to either stimulate proliferation of T regulatory cells and/or the release of the anti-inflammatory cytokine IL-10 to inhibit an inflammatory response [27]. Other GPRs (GPR-41, GPR-109A, etc) have been identified as ligands for SCFAs and SCFAs have been reported to have other intestinal protective effects (increased mucous production and stimulation of tight junction proteins) [9].

When we examined receptor expression in the fetal enterocyte cell line, we showed the GPR-43 and GPR41 mRNA was expressed at a very low level whereas GPR-109A mRNA had an increased expression (**Fig 7**) suggesting that this receptor was involved in SCFA-receptor interaction in the immature enterocyte. These observations were further supported when the SCFA anti-inflammatory effect was lost if cells were pretreated with the GPR-109A inhibitor mepenzolate bromside (MB) before an IL-1β stimulus (**Fig 8**). These observations suggest that the mechanism of anti-inflammation by SCFAs may differ between immature and mature enterocytes.

Another effect of SCFAs on mature enterocytes is that they are taken up by these cells and act as an inhibitor of HDACs which affect transcription of inflammatory cytokines such as IL-8, etc. in an epigenetic manner after an inflammatory stimulus [14,28]. In these studies, we showed that SCFAs alone had no effect on mRNA expression of HDAC3 or HDAC5 (**Fig 4**) but propionate and butyrate alone (not acetate) can inhibit the total HDACs protein expression **(Fig 5)** suggesting that propionate or butyrate alone inhibiting HDACs expression may act in an epigenetic manner. Furthermore, all three SCFAs can inhibit IL-1β induced HDAC3 and HDAC5 mRNA and HDACs protein in H4 cells (**Figs 4 and 5**) but not in a mature enterocyte cell line -Caco2 cells [29] (IL-1β could not stimulate HDAC3 and HDAC5 mRNA induction in Caco2 cells although IL-1β can stimulate IL8 induction in Caco2 cells) (**S1 Fig**) again suggesting a different mechanism for anti-inflammation between mature and immature enterocytes. The Caco-2 cell line is originally derived from a colon carcinoma. However, one of its most significant properties is its ability to spontaneously differentiate into a monolayer of cells with many properties typical of mature absorptive enterocytes with a brush border layer similar to the small intestine. Currently there is no normal human mature small intestinal cell line available. That is why Caco2 cells are used as a mature small intestinal epithelial cell line in many publications. So, in this study we used Caco2 cell line to mimic a normal adult small intestinal epithelial cell line [29].

In summary, this study suggests another mechanism for the synbiotic effect of expressed breast milk interacting with probiotics fed to premature infants in preventing excessive inflammation leading to necrotizing enterocolitis. The study also suggests that the anti-inflammatory effect of SCFAs in the immature intestine differs from the mechanism in the mature intestine. In order to determine other potential enterocyte mechanisms of immune protection by SCFAs, we plan to do transcription profiles of RNA from H4 cells after exposure to SCFAs and an inflammatory stimulus. Furthermore, if these initial observations are confirmed by a single protocol, multicenter trial with expressed breast milk and an established NEC probiotic, this may represent a future general treatment approach to preventing NEC in all premature infants at risk.

## Materials and methods

### Chemicals

Dulbecco’s modified Eagles medium (DMEM), nonessential amino acid (NEAA, 100×), glutamine (100×), antibiotic/antimycotic solution (100×), HEPES buffer (1 M) and sodium pyruvate (100 mM) were obtained from Gibco (ThermorFisher Scientific, San Diego, CA). Fetal bovine serum (FBS) was obtained from Atlanta Biologicals (Lawrenceville, GA). Humulin R regular insulin human injection material (100 units/ml) was obtained from Lilly (Indianapolis, IN). Tissue culture plastics were obtained from Fisher Scientific (Pittsburgh, PA). The RNAeasy Mini kits were obtained from Qiagen (Valencia, CA). SuperScript III first-strand synthesis supermix and SYBR GreenER qPCR SuperMix Universal were obtained from Life Technologies (Grand Island, NY). Recombinant human IL-1β and enzyme-linked immunosorbent assay (ELISA) kits for human IL-8 were obtained from R&D Systems (Minneapolis, MN). Short chain fatty acids (sodium acetate, sodium propionate and sodium butyrate) and G-coupled protein receptor (GPR) 109A inhibitor mepenzolate bromide and HDAC3 inhibitor Trichostain A (TSA) were purchased from Sigma Aldrich (Natick, MA). HDAC5 inhibitor LMK325 was purchased from Santa Cruz.

### Cell cultures and treatments

H4 cells, a human non-transformed primary intestinal epithelial cell line characterized by our laboratory (IRB 2018-P002987), were used as an in vitro model of the immature intestine [30]. The cells were routinely maintained in DMEM supplemented with 10% heat-inactivated FBS, 1% NEAA, 1% glutamine, 1% antibiotic/antimycotic solution, 10 mM HEPES buffer, 1 mM sodium pyruvate and 0.2 units/ml human recombinant insulin. Cells were incubated at 37°C in a 5% carbon dioxide, humidified atmosphere. Caco-2 cells were obtained from the American Type Culture Collection (ATCC, Manassas, VA) and cultured in DMEM with 10% FBS, 2mM L-glutamine, 0.1 mM MEM nonessential amino acids, 10 mM Hepes buffer, 100 units/ml penicillin and 100 μg/ml streptomycin. H4 or Caco2 cells were incubated without or with acetate (5−30 mM), propionate (5−30 mM), or butyrate (5−30 mM) in the absence or presence of 1 ng/ml IL-1β. IL-1β was added to cell culture medium 30 min after the administration of SCFAs

### Organoid cultures

Human sample procedures were approved by institutional review board protocols IRB 1999P003833 (Brigham and Women’s Hospital, Boston, MA) and IRB 2016P000949 (Massachusetts General Hospital, Boston, MA) for the derivation of fetal enterospheres (FEnS). The human fetal intestinal organoids were derived as described in our previous publication [20]. Briefly, intestinal fragments were collected from aborted fetuses and cut into small pieces. Crypt cells or H4 cells in the passage of 20 cultured in 150 cm^2^ flask to confluence were isolated with EDTA methods and the solutions containing intestinal crypts or H4 cells were processed further and plated in Matrigel (2x10^5^ cells in 11.2μl Matrigel and separated into 10 small tents per well in a 6- well plate) as described [20] to produce FEenS or H4 organoids respectively. Stem cell media was prepared according to our previously published methods [20]. The intestinal stem cell media composition was as follows: 500 mL Dulbecco’s modified Eagle medium (DMEM)/F12 11330–032, 5 mL penicillin/streptomycin 15140122, 5 mL nonessential amino acids 11140–050, 5 mL sodium pyruvate 11360–070, 5 mL N-2 17502, and 10 mLB-27 17504044 (all purchased from ThermoFisher Scientific); 50 mL fetal bovine serum F4135, 1 mmol/L acetylcysteine A9165, and 10 nmol/L gastrin G9145 (all purchased from Sigma-Aldrich); 50 ng/mL epidermal growth factor (EGF) (AF-100-15; PeproTech, Rocky Hill, NJ). L-WRN mouse fibroblasts ATCC CRL-3276 conditioned media was prepared as previously described. Stem cell media and L-WRN–derived conditioned media were combined in a 1:1 ratio (1:1 media), 500 nmol/L A-8301 SML0788-5MG (Sigma-Aldrich), 10 mmol/L Y27623 Y0503- 5MG (Sigma-Aldrich) were added before use. In this experiment the FEnS we used was from gestational age 22 week and 15 week, passage 17–20 therapeutically aborted fetuses.

Single-cell suspensions derived from organoids were plated on Polyester (PET) membrane Transwell inserts with a 0.4-mm pore size (Corning Life Sciences, Corning, NY) at 1.06 cells/mL. The media was changed every other day. When the culture reached confluence, based on transepithelial electrical resistance (TEER) monitoring and microscope direct observation (approximately 10 days), the culture was apically treated with 5 μmol/L N-[(3,5-Difluorophenyl) acetyl]-L-alanyl-2-phenyl]glycine-1, 1-dimethylethyl ester (DAPT) in DMEM/F12 for 48 hours as previously described to promote cell differentiation. All inhibitors were removed from 1:1 media on the basolateral side. The monolayers were treated with short chain fatty acid (20 mM) apically for 30 mins before being stimulated with human IL-1β (1ng/ml for 24 hours) basolaterally. The supernatants from the basolateral side were collected for IL-8 protein assay by ELISA.

### Determination of the anti-inflammatory effects of SCFAs on fetal and adult mouse intestinal organ culture

C57BL/6J (Jackson Laboratory) were bred and housed in a specific pathogen free facility. Animals were given water and standard laboratory chow *ad libitum*. Timed pregnant mice were established by pairing 12-14-wk old female mice with proven breeder male just prior to the end of the daily light cycle. The following morning the male was separated from the female. The females were placed in a dated cage (an examination for the presence of an ejaculatory plug in the vagina was omitted to reduce the perturbations to the mice) and considered pregnant, e.g., embryonic day (E) 0.5. Pregnant mice were confirmed when an enlarged abdomen became visually apparent near E18.5. Pups were delivered by caesarean section at E 18.5[22]. Small and large intestinal tissues were then collected and cut into 3-mm pieces and maintained in organ culture media as described previously [22]. Briefly, this media was made by Opti-MEM I medium supplemented with 10% FBS, 2 mM L-glutamine, 10 mM HEPES buffer, 0.2U/ml insulin, and 20 ng/ml recombined mouse epidermal growth factor (EGF) (R & D systems, Minneapolis, MN), 5 μg/ml transferrin, 0.06 μM sodium selenite, 200 nM hydrocortisone (Sigma, St. Louis, MO) and an antibiotic-antimycotic cocktail (100 unit/ml penicillin, 100 μg/ml streptomycin and 0.25 μg/ml fungizone antimycotic) (GibcoThermofisher). After 1–2 h at 37°C, tissues were pretreated with and without 20 mM of short chain fatty acids (acetate, propionate and butyrate) at 20 mM for 30 min and then stimulated with 1ng/ml of recombinant mouse IL-1β (R&D Systems, Minneapolis, MN) for 24 hrs. Supernatants were collected and stored at −20°C for ELISA analysis. Animal procedures had been previously approved by the Massachusetts General Hospital Subcommittee on Research Animal Care and Use committee (2018N000070).

### Measurement of IL-8 and macrophage inflammatory protein2(MIP2) production

IL-8 (human) and MIP2(murine homologue of IL8) concentrations in cell or tissue culture media were determined by ELISA with Human CXCL-8/IL-8 and Mouse CXCL2/MIP-2 DuoSet ELISA kits (R&D systems, Minneapolis, MN, USA) according to the manufacturer’s protocol. Absorbance at 450 nm was measured using a microplate spectrophotometer (Multiskan Go, Thermo Fisher Scientific Inc.). The total protein was measured by using Quick Start Bradford 1xDye Reagent (Bio-Rad, Hercules, California). IL-8 concentrations were normalized by total protein level, expressed as pg/mg protein.

### RNA isolation and quantitative RT-PCR analysis

Total RNA was extracted from H4 cells and H4 organoids with RNA RNeasy MIini Kit (Qiagen, Valencia, CA). Isolated RNA was treated with DNase I (Fermentas) to remove remaining DNA. RNA yield was quantified using a NanoDrop ND-1000 spectrophotometer (Thermo Scientific, Rockford, IL, USA). The cDNA was amplified using iQ SYBR Green Supermix (Bio-Rad, Philadelphia, PA). For quantitative RT-PCR of humanHDAC3,HDAC5, FFAR2, FFAR3, GRP109A and GADPH, and the primer sequences used of these genes were shown in below. The RT-PCR with probes was performed with the One Step RT-PCR Master-Mix kit for Probe Assays (Eurogentec) with 200 ng of RNA per reaction and 0.5 μM of primers and probes. Transcript levels were corrected to GADPH, and fold expression levels of gene of interests were calculated by their Ct value. Unstimulated condition: ΔCt control = Ct target gene control − Ct GADPH control. Stimulated condition: ΔCt treated = Ct target gene treated − Ct GADPH treated. ΔCt(control) − ΔCt(treated) values = ΔΔCt, the fold change in mRNA = 2^ΔΔCt^.

Human GAPDH, forward, 5’- ATGGGGAAGGTGAAGGTCG-3’,

and reverse, 5’- GGGGTCATTGATGGCAACAATA-3’

Human HDAC3, forward, 5’- GCAAGGCTTCACCAAGAGTCT-3’,

and reverse, 5’- AGATGCGCCTGTGTAACGC-3’

Human HDAC5, forward, 5’- TCTTGTCGAAGTCAAAGGAGC-3’,

and reverse, 5’- GAGGGGAACTCTGGTCCAAAG-3’

Human GPR109A, forward, 5’- ATGTTGGCTATGAACCGCCAG -3’,

and reverse, 5’- GCTGCTGTCCGATTGGAGA -3’

Human FFAR2, forward, 5’-CTTCGGACCTTACAACGTGTC-3’,

and reverse, 5’-CTGAACACCACGGCTATTGAC-3’

Human FFAR3, forward, 5’- TTCACCACCATCTATCTCACCG-3’,

and reverse, 5’-GGAACTCCAGGTAGCAGGTC-3’

### HDAC protein assay

HDAC activity was measured using the HDAC-Glo assay (Promega) according to the manufacturer’s instructions. H4 cells were seeded into a white-walled 96-well plate at a density of 10,000 cells/well in a volume of 50 μl for 72 h. H4 cells were pretreated with for SCFAs 30 min and then added with IL-1β for 24 h. Remove culture medium and replaced by serum-free medium. Subsequently, 50 μl of TSA was transferred on to the plate and incubated for 30 min and 100 μl HDAC-Glo^™^ I/II reagent was added to each well. The assay was incubated at room temperature for 20 min after which luminescence was measured using a Fluorostar plate reader (GE Healthcare).

### H4 cell line subjected to HDAC3, HDAC5 and GPR 109A inhibitor

To investigate the function of HDAC3, HDAC5 and GPR109A in the SCFAs IL-1 β-induced inflammation in H4 cells, H4 cells were pretreated with the HDAC3 inhibitor TSA(100 nM), HDAC5 inhibitor LMK325 (100 nM) and 109A inhibitor mepenzolate bromide(MP) [23] (0.2 mM dissolved in DMSO) for 30 min before SCFAs (20 mM of acetate, propionate and butyrate) treatment for 30 min then exposed to 1ng/ml of recombinant human IL-1β (R&D Systems) for 24 h. Supernatants were collected and stored at -20°C for ELISA IL8 analysis.

### Statistical analysis

All data are presented as the mean ±standard error of the mean (SEM). One-way ANOVA and Turkey post-doc test were used to compare the mean of multiple groups. Differences of *p* < 0.05 were considered significant (comparison with control group*p < 0.05, **p < 0.01, ***p < 0.0001; comparison with IL-1β treated group ^#^*p* < 0.05, ^##^*p* < 0.01, ^###^*p* < 0.0001) (GraphPad Prism 6).

## Supporting information

S1 Checklist The ARRIVE guidelines checklist. (PDF)

S2 Checklist The ARRIVE guidelines checklist. (DOCX)

S1 Fig SCFAs inhibited IL-1β induced IL8 induction in Caco2 cells but not by inhibition of HDACs activity. Caco2 cells were pretreated with or without 20 mM of SCFAs for 30 min before IL-1β stimulation (1ng/ml) for 24h **(A)** or 4h **(B-C)**. The secretion of IL-8 into the supernatants was assayed by Elisa **(A)** and the HDAC3 **(B)** and HDAC5 mRNA **(C)** were determined by real time RT-PCR. Data are represented as the mean ± SEM of three independent experiments, n = 3. One -way ANOVA and Tukey post hoc tests were used for statistic. Differences compared to the control group were considered significant at **p<0.01, *** p<0.001; differences compared to the IL-1β group were considered significant at ### p<0.001.
(TIF)

S1 Data (PDF)

## References

[1] Neu J, Walker WA. Necrotizing enterocolitis. N Engl J Med. 2011; 364: 255–264. 10.1056/NEJMra1005408 21247316 PMC3628622

[2] Nanthakumar N, Meng D, Goldstein AM, Zhu W, Lu K, Uauy R, et al The mechanism of excessive intestinal inflammation in necrotizing enterocolitis: An immature innate immune response. Plos One. 2011; 6:(3): e17776–e17786. 10.1371/journal.pone.0017776 21445298 PMC3061868

[3] Claud EC, Lu L, Anton PM, Savidge T, Walker WA, Cherayil BJ. Developmentally-regulated IL-1 β expression in intestinal epithelium and susceptibility to flagellin-induced inflammation. PNAS. 2004; 101:7404–7408. 10.1073/pnas.0401710101 15123821 PMC409931

[4] Nanthakumar N, Fusunyan RD, Sanderson IR, Walker WA. Inflammation in the developing human intestine: A possible pathophysiologic basis for necrotizing enterocolitis. PNAS. 2000; 97:6043–6048. 10.1073/pnas.97.11.6043 10823949 PMC18555

[5] Nanthakumar NN, Young C, Ko JS, Meng D, Chen J, Buie T, et al Glucocorticoid responsiveness in the developing human intestine: possible role in the prevention of necrotizing enterocolitis. AJP: GI and Liver Physiology. 2005; 288: G85–G92. 10.1152/ajpgi.00169.2004 15591589

[6] Walker WA, Iyengar RS. Breast milk, microbiota and intestinal immune homeostasis. Pediatr Res. 2015; 77:220–228. 10.1038/pr.2014.160 25310762

[7] Ganguli K, Walker WA. Probiotics in the prevention of necrotizing enterocolitis. J Clin Gastroenterology. 2011; 45: S133–S138. 10.1097/MCG.0b013e318228b799 21992952

[8] Repa A, Thanhaeuser M, Endress D, et al Probiotics (Lactobacillus acidophilus and Bifidobacterium infantis) prevent NEC in VLBW infants fed breast milk but not formula. Pediatric Research. 2015;77(2):381–388. 10.1038/pr.2014.192 25423074

[9] Tan J, McKenzie C, Potamitis M, Thorburn AN, Mackay CR, Macia L. Chapter Three—The Role of Short-Chain Fatty Acids in Health and Disease. Advances in Immunology. 2014; 121:91–119. 10.1016/B978-0-12-800100-4.00003-9 24388214

[10] Jiang Z, Liu Y, Zhu Y, et al Characteristic chromatographic fingerprint study of short-chain fatty acids in human milk, infant formula, pure milk and fermented milk by gas chromatography-mass spectrometry. International Journal Of Food Sciences And Nutrition. 2016;67(6):632–640. 10.1080/09637486.2016.1195798 27282191

[11] Pourcyrous M, Nolan VG, Goodwin A, Davis SL, Buddington RK. Fecal short-chain fatty acids of very-low-birth-weight preterm infants fed expressed breast milk or formula. J Pediatr Gastroenterol Nutr. 2014;59(6):725–31. 10.1097/MPG.0000000000000515 25079478

[12] Yu ZT, Chen C, Newburg DS. Utilization of major fucosylated and sialylated human milk oligosaccharides by isolated human gut microbes. Glycobiology. 2013;23(11):1281–92. 10.1093/glycob/cwt065 24013960 PMC3796377

[13] Maslowski KM, Vieira AT, Ng A, et al Regulation of inflammatory responses by gut microbiota and chemoattractant receptor GPR43. Nature. 2009;461(7268):1282–1286. 10.1038/nature08530 19865172 PMC3256734

[14] Huda-Faujan N, Abdulamir AS, Fatimah AB, et al The impact of the level of the intestinal short chain fatty acids in inflammatory bowel disease patients versus healthy subjects. The Open Biochemistry Journal. 2010; 4:53–58. 10.2174/1874091X01004010053 20563285 PMC2887640

[15] Marchesi JR, Holmes E, Khan F, Kochhar S, Scanlan P, Shanahan F, et al Rapid and noninvasive metabonomic characterization of inflammatory bowel disease. J Proteome Res. 2007; 6:546–551. 10.1021/pr060470d 17269711

[16] Lin MY, Marcel R. de Zoete, Jos P. M. van Putten and Karin Strijbis. Redirection of epithelial immune responses by short-chain fatty acids through inhibition of histone deacetylases. Front Immunol. 2015; 03(6): Article 554. 10.3389/fimmu.2015.00554 PMC4630660 26579129

[17] Fellows R, Denizot J, Stellato C, Cuomo A, Jain P, Stoyanova E, et al Microbiota derived short chain fatty acids promote histone crotonylation in the colon through histone deacetylases. Nat Commun. 2018;9(1):105 10.1038/s41467-017-02651-5 29317660 PMC5760624

[18] Li M, van Esch B, Wagenaar GTM, Garssen J, Folkerts G, Henricks PAJ. Pro- and anti- inflammatory effects of short chain fatty acids on immune and endothelial cells. Eur J Pharmacol. 2018;831:52–9. 10.1016/j.ejphar.2018.05.003 29750914

[19] Macia L, Thorburn AN, Binge LC, et al Microbial influences on epithelial integrity and immune function as a basis for inflammatory diseases. Immunological Reviews. 2012; 245:164–176. 10.1111/j.1600-065X.2011.01080.x 22168419

[20] Senger S, Ingano L, Freire R, Anselmo A, Zhu W, Sadreyev R, et al Human fetal-derived enterospheres provide insights on intestinal development and a novel model to study Necrotizing Enterocolitis (NEC). Cellular and Molecular Gastroenterology and Hepatology Journal 2018; 5:549–568. 10.1016/j.jcmgh.2018.01.014 PMC6009798 29930978

[21] Weng M, Ganguli K, Zhu W, Shi HN, Walker WA. Conditioned media from *Bifidobacteria infantis* protects against *Cronobacter sakzakii-*induced intestinal inflammation in newborn mice. Am J Phys- Gastrointestinal and Liver Physiol. 2014; 306: G779–G787. 10.1152/ajpgi.00183.2013 PMC4010653 24627567

[22] Meng D, Zhu W, Shi HN, Lu L, Wijendran V, Xu W, et al The toll-like receptor -4 in human and mouse colonic epithelium is developmentally regulated: a possible role in necrotizing enterocolits. Pediatr Res. 2015; 77:416–24. 10.1038/pr.2014.207 25521917 PMC4479150

[23] Tanaka KI, Yamakawa N, Yamashita Y, Asano T, Kanda Y, Takafuji A, et al Identification of Mepenzolate Derivatives With Long-Acting Bronchodilatory Activity. Front Pharmacol. 2018; 9:344 10.3389/fphar.2018.00344 29692733 PMC5902689

[24] Walker WA. The importance of appropriate initial bacterial colonization of the intestine in newborn, child and adult health. Pediatric Research. 2017:82:387–395. 10.1038/pr.2017.111 28426649 PMC5570628

[25] O’Rourke L, Clarke G, Nolan A, et al Tryptophan metabolic profile in term and preterm breast milk: implications for health. Journal of Nutritional Science. 2018;7: e13–22. 10.1017/jns.2017.69 29686862 PMC5906556

[26] Bridgman S.L., Azad M.B., Field C.J., et al Fecal short-chain fatty acid variations by breastfeeding status in infants at 4 months: Differences in relative versus absolute concentrations. Front. Nutr. 2017; 4:11–24. 10.3389/fnut.2017.00011 28443284 PMC5385454

[27] Vangaveti V, Shashidhar V, Jarrod G, Baune BT, Kennedy RL. Free fatty acid receptors: emerging targets for treatment of diabetes and its complications. Ther Adv Endocrinol Metab. 2010;1(4):165–75. 10.1177/2042018810381066 23148161 PMC3474614

[28] Licciardi PV, Ververis K and Karagiannis TC. Histone deacetylase inhibition and dietary short-chain fatty acids. ISRN Allergy. 2011; Article 869647. 10.5402/2011/869647 PMC3658706 23724235

[29] Meunier V, Bourrié M, Berger Y, Fabre G. The human intestinal epithelial cell line Caco-2; pharmacological and pharmacokinetic applications. Cell biol toxicol. 1995;11(3–4):187–94. 10.1007/bf00756522 8564649

[30] Sanderson IR, Ezzell RM, Kedinger M, Erlanger M, Xu Z, Pringault E, et al Human fetal enterocytes *in vitro*: modulation of the phenotype by extracellular matrix. PNAS. 1996; 93:7717–7722. 10.1073/pnas.93.15.7717 8755542 PMC38813

