# Supplementary material for: Short chain fatty acids produced by colonizing intestinal commensal bacterial interaction with expressed breast milk are anti-inflammatory in human immature enterocytes
Source: PLoS One. 2020 Feb 21;15(2):e0229283. doi: 10.1371/journal.pone.0229283 (PMC7034856; doi:10.1371/journal.pone.0229283)
Supplement: S2 Checklist — (DOCX) [file pone.0229283.s002.docx]

**The ARRIVE Guidelines Checklist**

Animal Research: Reporting In Vivo Experiments

1. **Title**

**Short Chain fatty acids produced by colonizing intestinal commensal bacterial interaction with expressed breast milk are anti-inflammatory in human immature enterocytes**

1. **Abstract**

Necrotizing enterocolitis is a devastating intestinal emergency that affects ten percent of very low birth weight premature babies and costs society in both expense and heartache. It is probably caused by an inappropriate interaction of colonizing bacteria with an immature intestine. A possible preventative measure is to feed prematures their mother’s expressed breast milk in conjunction with a probiotic. This synbiotic prevention reduces the severity and incidence of this condition. This study was designed to determine the mechanism of the synbiotic effect in human and mouse fetal intestine. Breast milk interacting with a NEC preventative probiotic such as *Bifidobacteria infantis* can produce increased levels of short chain fatty acids (acetate, propionate and butyrate) (SCFAs). SCFAs are known to be anti-inflammatory in mature enterocytes and immunocytes. Very little is known about their role in immature intestine. When exposed to a human fetal cell line, fetal intestinal organoids and **fetal mouse (C57BL/6J, E18.5)** **intestinal organ culture**, these SCFAs were anti-inflammatory. Their mechanism of anti-inflammation differed from those reported for mature cells by involving the G-protein coupled receptor (GPR 109A) and inhibiting histone deacetylase 4 and 5. These bacterial metabolites may help explain the synbiotic anti-inflammatory effect of breast milk and probiotics given to premature infants at risk for NEC.

1. **Background**

Necrotizing enterocolitis (NEC) is an inflammatory necrosis of the distal small intestine and colon that commonly affects very premature infants (10% incidence) less than 1500grams in birthweight (1). We have reported that its pathogenesis is in part due to an inappropriate reaction of colonizing bacteria with an immature intestine (2). For example, colonizing bacteria (both pathogens and commensals) can create an excessive inflammatory response in the immature intestine rather than developing immune homeostasis (3). This excessive inflammatory response may be due to an immature innate reaction to the colonizing bacteria (2,4). A recent study suggests that prevention is most effective when breast milk and probiotics are given together (synbiotic effect) (5). Short chain fatty acids (SCFAs) are produced by intestinal commensal bacteria interacting with a diet rich in complex carbohydrates which cannot be metabolized by enzymes in the small intestine (6). In the immature human intestine, SCFAs are produced when expressed breastmilk fed to prematures interacts with colonizing bacteria (7,8)**.** In this study, we determined if SCFAs (acetate, propionate and butyrate) were anti-inflammatory in a fetal cell line, in fetal organoids and in fetal mouse intestine after an inflammatory stimulus with IL-1β. Accordingly, this study was designed to investigate how the synbiotic effect works by determining the interaction of bacterial metabolites [short chain fatty acids (SCFAs)] on immature intestinal inflammation.

Because mouse is biologically similar to human therefore in this study, we detected the anti-inflammatory effects of SCFAs on whole intestinal tissue from C57BL6 fetal mouse (embryonic day 18.5) with organ culture.

1. **Objectives**

Determine the effect of bacterial metabolites [short chain fatty acids (SCFAs)] on immature intestinal inflammation.

1. **Ethical statement:** Animal procedures had been previously approved by the Massachusetts General Hospital Subcommittee on Research Animal Care and Use committee (2018N000070).
2. **Study design:** C57BL6/J fetal mice (embryonic day 18.5) small or large intestinal organ cultures were treated in the 48-well tissue culture plates as following:
3. Con
4. Acetate 20 mM
5. Propionate 20 mM
6. Butyrate 20 mM
7. IL-1β 1ng/ml for 24 h
8. Acetate 20mM for 30 min then IL-1β 1ng/ml for 24 h
9. Propionate 20mM for 30 min then IL-1β 1ng/ml for 24 h
10. Butyrate 20mM for 30 min then IL-1β 1ng/ml for 24 h

At the end of the experiments the supernatant was collected and kept at -20˚C for Microphage Inflammatory Protein (MIP2) assay with Elisa.

1. **Experimental procedures:** Timed pregnant mice were established by pairing 12-14-wk old female mice with proven breeder male just prior to the end of the daily light cycle. The following morning the male was separated from the female. The females were placed in a dated cage and considered pregnant, e.g., embryonic day (E) 0.5. Pregnancy was confirmed by detection of the belly enlarged visually apparent near E18.5. At the day of E 18.5 the compressed CO2 was used to euthanasia the pregnant mice and the cervical dislocation was used to confirm the death then the pups were delivered by caesarean section (mostly the pups don’t have breath, if they have breath the decapitation would be used as an euthanasia method). Fetal mice small and large intestinal tissues were then collected and cut into 3-mm pieces and maintained in organ culture media as described previously (9).
2. **Experimental animals:** C57BL6/J mice were originally purchased from Jackson Laboratory. 12-14-wk old female and male mice were bread as described above. E 18.5 fetal mice small and large intestinal organ culture were used to determine the anti-inflammatory effects of SCFAs. Two independent experiments were performed and 6 fetal mice in each experiment.
3. **Housing and husbandry:** Mice were bred and housed in a specific pathogen free facility in ventilated cages on a 12-h light/dark cycle. Animals were given water and standard laboratory chow *ad libitum.*
4. **Sample size**: In each animal experiment the sample size is 6 (fetal mice). We chose this size is based on the combination of the principle below:
5. At least sample size =3 can do statistical analysis
6. less number of animals it may lead to missing of any significant difference even if it exists in population, and if the more number of animals selected then it may lead to unnecessary wastage of resources and may lead to ethical issues.
7. Reference the literature (10) and our previous publication (9)
8. Pay attention to how much minimum difference between two groups can be considered as statistically significant and how big the deviation is.
9. Based on all the rational description above, we considered that 6 fetal mice for each experiment is the best for our study.
10. We repeated the experiment to avoid the deviation from the individual pregnant mice.
11. **Allocating animals to experimental groups:** We collected the small and large intestinal tissues from each fetal mouse and cut them to 0.3cm small pieces then randomly assigned them to different treatment in the 48-well plates.
12. **Experimental outcomes:** The primary and secondary experiment had the similar outcome, in which short-chain fatty acids (SCFAs) inhibited IL-1β induced microphage inflammatory protein2(MIP2) induction in fetal small and large intestinal organ cultures.
13. **Statistical methods:** All data are presented as the mean ±standard error of the mean (SEM). One-way ANOVA was used to compare the mean of multiple groups. Differences of *p* < 0.05 were considered significant (vis control group **p* < 0.05, ***p* < 0.01, ****p* < 0.0001) (vis IL-1β treated group ^#^*p* < 0.05, ^##^*p* < 0.01, ^###^*p* < 0.0001) (GraphPad Prism 6).
14. **Baseline data:** In this study, the animal experiments were ex vivo. There was no treatment before the euthanasia of the
    \mice.
15. **Numbers analysed:** 6 fetal mice in each group and 6 number in each analysis.
16. **Outcomes and estimation:** The results are presented as the mean ±standard error of the mean (SEM).
17. **Adverse events:** When we did fetal mouse intestinal tissue culture if the culture media is not enough, the cytotoxicity will be increased. For example, 1 ml media/tissue/well in a 48-well tissue culture plate has less cytotoxicity then 0.5 ml media/tissue/well. Cytotoxicity can be assed by LDH assay kit.
18. **Interpretation/scientific implications:** Our results showed that short-chain fatty acids (SCFAs) can inhibited IL-1β induced MIP2 induction in fetal mouse small and large intestinal organ cultures which confirmed the anti-inflammatory functions of SCFAs in immature monolayer epithelial cells in addition the animal results provided the new information that SCFAs also have anti-inflammatory effects on the whole intestinal tissue. The animal experiment results also took into account the study hypothesis that SCFAs have an anti-inflammatory function in immature intestine.

The limitation of the study is that this is an ex vivo study, in the future we plan to do the in vivo study to investigate the effect of the oral feeding of the SCFAs on inflammation in neonatal mice.

1. **Generalisability/translation:** Short-chain fatty acids (SCFAs) are the metabolites from the symbiosis interaction of breast milk and probiotics. It was reported that breast milk interacting with probiotics fed to premature infants could reduce the risk of the Necrotizing enterocolitis (NEC) in premature infants. NEC is the leading cause of the death in gastrointestinal disease in premature infants and is an uncurable disease. Our finding will provide the new therapeutic strategy for NEC.

**20. Funding:** This study was supported by the following grants.

-NIDDK (P01-DK033506) “Barrier function of the GI tract in health and disease”

PI W. Allan Walker

-Family Larsson-Rosenquist Foundation “The impact of breastmilk microbiome and protective

factors on development of human intestinal disease” PI W. Allan Walker

-Beth Israel/Deaconess Medical Center (Award #01027741) “Impact of breastmilk in

premature intestinal colonization” PI W. Allan Walker

**Reference:**

1. Neu J, Walker WA. Necrotizing enterocolitis. N Engl J Med; 2011; 364: 255-264.
2. Nanthakumar N, Meng D, Goldstein AM, Zhu W, Lu K, Uauy R, Llano A, Claud EC, Walker WA. The mechanism of excessive intestinal inflammation in necrotizing enterocolitis: An immature innate immune response. Plos One 2011; 6:(3):e17776-e17786.
3. Claud EC, Lu L, Anton PM, Savidge T, Walker WA, Cherayil BJ. Developmentally-regulated IL-1 β expression in intestinal epithelium and susceptibility to flagellin-induced inflammation. PNAS 2004; 101:7404-7408.
4. Nanthakumar N, Fusunyan RD, Sanderson IR, Walker WA. Inflammation in the developing human intestine: A possible pathophysiologic basis for necrotizing enterocolitis. PNAS 2000; 97:6043-6048.
5. Repa A, Thanhaeuser M, Endress D, et al. Probiotics (Lactobacillus acidophilus and Bifidobacterium infantis) prevent NEC in VLBW infants fed breast milk but not formula. Pediatric Research. 2015;77(2):381-388.
6. Tan J, McKenzie C, Potamitis M, Thorburn AN, Mackay CR, Macia L. Chapter Three - The Role of Short-Chain Fatty Acids in Health and Disease. Advances in Immunology. 2014;121:91-119.
7. Jiang Z, Liu Y, Zhu Y, et al. Characteristic chromatographic fingerprint study of short-chain fatty acids in human milk, infant formula, pure milk and fermented milk by gas chromatography-mass spectrometry. International Journal Of Food Sciences And Nutrition. 2016;67(6):632-640.
8. Pourcyrous M, Nolan VG, Goodwin A, Davis SL, Buddington RK. Fecal short-chain fatty acids of very-low-birth-weight preterm infants fed expressed breast milk or formula. J Pediatr Gastroenterol Nutr. 2014;59(6):725-31.
9. Meng D, Zhu W, Shi HN, Lu L, Wijendran V, Xu W, Walker WA. The toll-like receptor -4 in human and mouse colonic epithelium is developmentally regulated: a possible role in necrotizing enterocolits. Pediatr Res 2015; 77:416-24.
10. Charan J, Kantharia ND. How to calculate sample size in animal studies?. *J Pharmacol Pharmacother*. 2013;4(4):303–306. doi:10.4103/0976-500X.119726
