# Supplementary figures and images for: Short chain fatty acids produced by colonizing intestinal commensal bacterial interaction with expressed breast milk are anti-inflammatory in human immature enterocytes
Source: PLoS One. 2020 Feb 21;15(2):e0229283. doi: 10.1371/journal.pone.0229283 (PMC7034856; doi:10.1371/journal.pone.0229283)

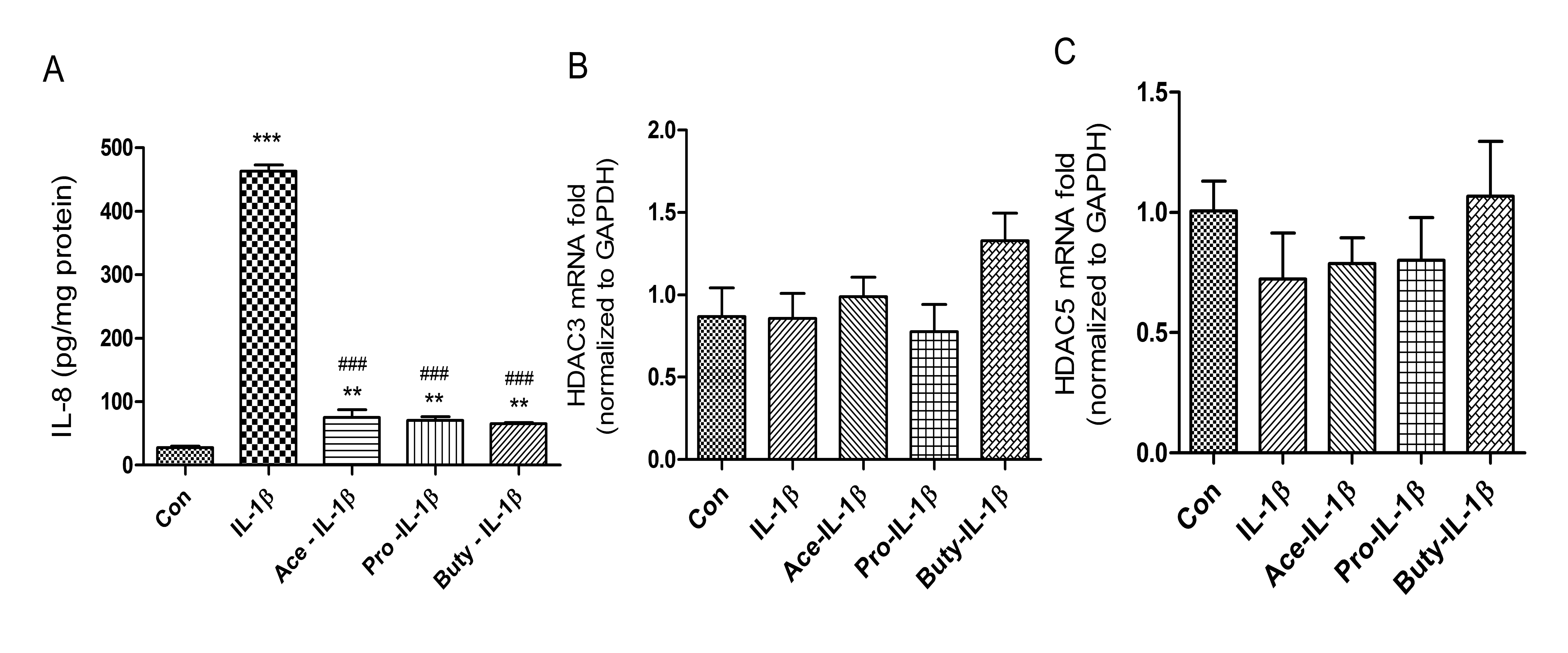

Supplement: S1 Fig — Caco2 cells were pretreated with or without 20 mM of SCFAs for 30 min before IL-1β stimulation (1ng/ml) for 24h (A) or 4h (B-C). The secretion of IL-8 into the supernatants was assayed by Elisa (A) and the HDAC3 (B) and HDAC5 mRNA (C) were determined by real time RT-PCR. Data are represented as the mean ± SEM of three independent experiments, n = 3. One -way ANOVA and Tukey post hoc tests were used for statistic. Differences compared to the control group were considered significant at **p<0.01, *** p<0.001; differences compared to the IL-1β group were considered significant at ### p<0.001. (TIF) [file pone.0229283.s003.tif]
