# Supplementary material for: Short chain fatty acids produced by colonizing intestinal commensal bacterial interaction with expressed breast milk are anti-inflammatory in human immature enterocytes
Source: PLoS One. 2020 Feb 21;15(2):e0229283. doi: 10.1371/journal.pone.0229283 (PMC7034856; doi:10.1371/journal.pone.0229283)
Supplement: S1 Data — (PDF) [file pone.0229283.s004.pdf]

Fig 1.The effects of different doses of short-chain fatty acids (SCFAs) on prevention of IL-1β-induced IL-8 induction in H4 cells.

A	Con	IL-1β	Ace 5mM-IL-1β	Ace 10mM-IL-1β	Ace 20mM-IL-1β	Ace 30mM-IL-1β
IL8(pg/mg protein)	52.825	1453.376	965.813	791.379	258.939	76.022
	55.575	1211.505	944.436	817.771	271.825	81.368
	60.497	1475.066	950.636	795.051	308.373	95.882
**Average**	**56.299**	**1379.982**	**953.628**	**801.400**	**279.712**	**84.424**
SD	3.887	146.308	10.998	14.296	25.644	10.277

B	Con	IL-1β	Pro 5mM-IL-1β	Pro 10mM-IL-1β	Pro 20mM-IL-1β	Pro 30mM-IL-1β
IL8(pg/mg protein)	52.825	1453.376	705.500	748.230	496.256	382.263
	55.575	1211.505	638.961	674.413	535.368	441.531
	60.497	1475.066	820.252	599.458	458.374	449.388
**Average**	**56.299**	**1379.982**	**721.571**	**674.034**	**496.666**	**424.394**
SD	3.887	146.308	91.708	74.387	38.498	36.697

C	Con	IL-1β	Buty 5mM-IL-1β	Buty 10mM-IL-1β	Buty 20mM-IL-1β	Buty 30mM-IL-1β
IL8(pg/mg protein)	52.825	1453.376	1075.495	621.582	729.811	742.070
	55.575	1211.505	1213.957	442.900	666.235	682.192
	60.497	1475.066	1393.093	991.664	642.199	658.468
**Average**	**56.299**	**1379.982**	**1227.515**	**685.382**	**679.415**	**694.243**
SD	3.887	146.308	159.232	279.890	45.268	43.084

Fig 2. SCFA inhibited IL-1β induced IL-8 secretion in H4 organoids and fetal human intestinal organoids.

A	Con	IL-1β	Ace-IL-1β	Pro-IL-1β	Buty-IL-1β
IL8(pg/mg protein)	3877.257	9801.792	5787.86	3931.875	5470.684
	3791.802	9906.25	5534.542	4660.213	5457.261
	3209.186	7542.541	4679.091	5392.119	5049.431
**Average**	**3626.082**	**9083.528**	**5333.831**	**4661.402**	**5325.792**
SD	363.562	1335.555	580.996	730.123	239.430

B	Con	IL-1β	Ace-IL-1β	Pro-IL-1β	Buty-IL-1β
IL8(pg/mg protein)	348.719	451.427	396.819	275.035	296.982
	348.557	486.971	427.190	263.215	325.936
	376.641	578.029	385.319	244.607	269.337
**Average**	**357.972**	**505.476**	**403.110**	**260.952**	**297.418**
SD	16.167	65.298	21.632	15.340	28.302

C	Con	IL-1β	Ace-IL-1β	Pro-IL-1β	Buty-IL-1β
IL8(pg/mg protein)	928.992	2479.371	1094.093	1180.704	1490.306
	1050.068	1916.334	1106.584	1052.224	1447.706
	1031.451	1966.013	1094.093	948.612	1246.876
**Average**	**1003.504**	**2120.573**	**1098.257**	**1060.513**	**1394.963**
SD	65.197	311.720	7.212	116.268	130.004

Fig 3. The anti-inflammatory effects of SCFAs on immature enterocytes in ex vivo.

A	Con	IL-1β	Ace-IL-1β	Pro-IL-1β	Buty-IL-1β
**MIP2**	28.932	388.940	37.909	62.260	33.761
**(pg/mg protein)**	22.033	322.404	49.815	43.465	53.640
	51.375	354.497	77.106	63.148	83.612
	63.766	357.779	96.218	63.468	82.878
	98.350	260.631	70.979	82.729	75.646
	113.639	361.347	80.471	86.741	65.649
**Average**	**63.016**	**340.933**	**68.750**	**66.968**	**65.865**
SD	36.847	44.679	21.337	15.753	19.392
**B**	**Con**	**IL-1β**	**Ace-IL-1β**	**Pro-IL-1β**	**Buty-IL-1β**
**MIP2**	31.001	349.426	68.460	79.776	80.605
**(pg/mg protein)**	29.272	304.590	70.904	78.532	77.645
	32.411	281.947	68.277	74.888	66.943
	27.835	272.426	64.093	62.249	47.281
	31.772	255.415	67.033	85.176	47.761
	19.533	274.758	65.687	60.516	48.153
**Average**	**28.637**	**289.760**	**67.409**	**73.523**	**61.398**
SD	4.766	33.305	2.374	9.981	15.648

Fig 4. SCFAs inhibit IL-1β induced histone deacetylases 3 (HDAC3) and HDAC5 mRNA expression in immature enterocytes.

A	Con	Ace	Pro	Buty	IL-1β	Ace-IL-1β	Pro-IL-1β	Buty-IL-1β
**H4-HDAC3 mRNA fold**	0.992	0.757	1.924	1.940	8.360	3.001	3.079	0.748
(Normalized to GAPDH**)**	0.906	1.342	2.107	1.562	11.349	3.970	4.423	0.872
	1.112	1.137	1.801	1.661	9.457	3.034	3.535	1.035
**Average**	**1.004**	**1.079**	**1.944**	**1.721**	**9.722**	**3.335**	**3.679**	**0.885**
SD	0.103	0.297	0.154	0.196	1.512	0.550	0.683	0.144
**B**	**Con**	**Ace**	**Pro**	**Buty**	**IL-1β**	**Ace-IL-1β**	**Pro-IL-1β**	**Buty-IL-1β**
**H4-HDAC5 mRNA fold**	1.004	1.398	1.662	1.897	10.986	0.462	0.742	0.443
(Normalized to GAPDH)	0.919	1.867	1.651	1.310	12.553	0.480	0.747	0.654
	1.083	1.666	1.544	1.058	12.466	0.349	0.929	0.471
**Average**	**1.002**	**1.644**	**1.619**	**1.422**	**12.002**	**0.430**	**0.806**	**0.523**
SD	0.082	0.236	0.065	0.430	0.881	0.071	0.107	0.114

Fig 5. SCFAs inhibit IL-1β induced HDACs protein expression in immature enterocytes.

HDACs Protein	Con	Ace	Pro	Buty	IL-1β	Ace-IL-1β	Pro-IL-1β	Buty-IL-1β
(Luminescent intensity)	7416.0	7647.0	3633.0	3241.0	19115.0	3852.0	2469.0	3241.0
	6771.0	8255.0	4877.0	3063.0	20670.0	3707.0	2997.0	3063.0
	7594.0	6207.0	4013.0	2997.0	16651.0	4334.0	1189.0	4013.0
**Average**	**7260.3**	**7369.7**	**4174.3**	**3100.3**	**18812.0**	**3964.3**	**2218.3**	**3439.0**
SD	433.0	1051.8	637.5	126.2	2026.6	328.2	929.7	505.0

Fig 6. Both HDAC3 and HDAC5 are required for mediating IL-1β induced IL-8 secretion in immature enterocytes *in vitro* and *ex vivo*

A	Con	IL-1β	TSA-IL-1β	LMK-IL-1β
**IL8(pg/mg protein)**	68.618	1453.376	825.419	919.872
	69.027	1211.505	802.803	866.237
	74.170	1475.066	766.930	545.120
**Average**	**70.605**	**1379.982**	**798.384**	**777.076**
SD	3.094	146.308	29.494	202.662
**B**	**Con**	**IL-1β**	**TSA-IL-1β**	**LMK-IL-1β**
**MIP2 (pg/mg protein)**	46.896	160.702	88.374	35.974
	37.205	164.465	71.539	33.517
	29.076	160.975	58.002	34.400
	51.375	151.262	92.871	53.418
	63.766	147.962	55.967	52.571
	29.076	156.541	60.842	57.285
**Average**	**42.899**	**156.984**	**71.266**	**44.528**
SD	12.502	5.774	14.596	10.029
**C**	**Con**	**IL-1β**	**TSA-IL-1β**	**LMK-IL-1β**
**MIP2 (pg/mg protein)**	31.001	349.426	61.155	69.647
	29.272	304.590	50.734	70.888
	32.411	272.896	50.949	68.390
	35.023	281.946	67.238	69.647
	45.206	264.577	66.912	70.888
	44.090	222.465	75.584	68.390
**Average**	**36.167**	**282.650**	**62.095**	**69.642**
SD	6.843	42.391	9.857	1.117

Fig 7. SCFAs increase G protein-coupled receptors 109A (GPR109A) mRNA expression in response to IL-1β stimulation in human immature enterocytes

	**Con**	**Ace**	**Pro**	**Buty**	**IL-1β**	**Ace-IL-1β**	**Pro-IL-1β**	**Buty-IL-1β**
**109A mRNA fold**	1.16	2.88	2.94	4.16	2.39	16.46	10.97	10.01
(Normalized to GAPDH)	0.86	3.28	4.06	4.92	1.89	19.90	15.47	14.65
	1.00	3.14	4.54	4.29	2.31	22.15	10.86	15.75
**Average**	**1.01**	**3.10**	**3.85**	**4.46**	**2.20**	**19.50**	**12.43**	**13.47**
SD	0.152	0.202	0.821	0.409	0.267	2.866	2.631	3.047

Fig 8. 109A is required for SCFAs anti IL-1β induced IL8 induction in H4 cells

	**Con**	**IL-1β**	**0.2mM MB Ace-IL-1β**	**0.2mM MB Pro-IL-1β**	**0.2mM MB Buty -IL-1β**	**Ace-IL-1β**	**Pro-IL-1β**	**Buty-IL-1β**
**IL8 (pg/ mg protein)**	18.959	919.264	864.756	873.847	1440.781	585.205	396.026	547.326
	14.809	1371.54	904.844	945.696	916.526	642.623	459.94	684.226
	12.869	871.819	885.766	994.976	1324.138	627.615	336.368	426.11
**Average**	**15.546**	**1054.208**	**885.122**	**938.173**	**1227.148**	**618.481**	**397.445**	**552.554**
SD	3.111	275.840	20.052	60.914	275.256	29.779	61.798	129.137

Supporting information

S1 Fig. SCFAs inhibited IL-1β induced IL8 induction in Caco2 cells but not by inhibition of HDACs activity

A	Con	IL-1β	Ace-IL-1β	Pro-IL-1β	Buty-IL-1β
IL8(pg/mg protein) (Caco2)	23.521	483.126	97.785	70.813	64.532
	31.709	456.819	70.778	80.659	69.341
	28.072	449.254	57.274	60.260	61.358
**Average**	27.767	463.067	75.279	70.577	65.077
SD	4.102	17.779	20.627	10.201	4.019
**B**	**Con**	**IL-1β**	**Ace-IL-1β**	**Pro-IL-1β**	**Buty-IL-1β**
**Caco2 -HDAC3 mRNA fold**	1.067	0.717	1.008	0.929	1.138
(Normalized to GAPDH)	0.744	1.021	0.857	0.598	1.411
	0.794	0.831	1.095	0.802	1.440
**Average**	0.868	0.856	0.987	0.777	1.330
SD	0.174	0.153	0.120	0.167	0.166
**C**	**Con**	**IL-1β**	**Ace-IL-1β**	**Pro-IL-1β**	**Buty-IL-1β**
**Caco2-HDAC5 mRNA fold**	1.029	0.626	0.668	0.996	0.890
(Normalized to GAPDH)	0.870	0.601	0.815	0.646	0.984
	1.117	0.944	0.878	0.762	1.325
**Average**	1.005	0.724	0.787	0.801	1.066
SD	0.125	0.191	0.108	0.178	0.229
